# Chromosome-scale assemblies of *S. malaccense, S. aqueum, S. jambos*, and *S. syzygioides* provide insights into the evolution of *Syzygium* genomes

**DOI:** 10.3389/fpls.2023.1248780

**Published:** 2023-10-06

**Authors:** Sonia Ouadi, Nicolas Sierro, Felix Kessler, Nikolai V. Ivanov

**Affiliations:** ^1^ Faculty of Sciences, Laboratory of Plant Physiology, University of Neuchâtel, Neuchâtel, Switzerland; ^2^ Philip Morris International R&D, Philip Morris Products S.A., Neuchâtel, Switzerland

**Keywords:** *Syzygium*, Myrtaceae, *de novo* assembly, comparative genomics, synteny, long terminal repeat retrotransposons

## Abstract

*Syzygium* is a large and diverse tree genus in the Myrtaceae family. Genome assemblies for clove (*Syzygium aromaticum*, 370 Mb) and sea apple (*Syzygium grande*, 405 Mb) provided the first insights into the genomic features and evolution of the *Syzygium* genus. Here, we present additional *de novo* chromosome-scale genome assemblies for *Syzygium malaccense, Syzygium aqueum, Syzygium jambos*, and *Syzygium syzygioides.* Genome profiling analyses show that *S. malaccense*, like *S. aromaticum* and *S. grande*, is diploid (2n = 2x = 22), while the *S. aqueum*, *S. jambos*, and *S. syzygioides* specimens are autotetraploid (2n = 4x = 44). The genome assemblies of *S. malaccense* (430 Mb), *S. aqueum* (392 Mb), *S. jambos* (426 Mb), and *S. syzygioides* (431 Mb) are highly complete (BUSCO scores of 98%). Comparative genomics analyses showed conserved organization of the 11 chromosomes with *S. aromaticum* and *S. grande*, and revealed species-specific evolutionary dynamics of the long terminal repeat retrotransposon elements belonging to the Gypsy and Copia lineages. This set of *Syzygium* genomes is a valuable resource for future structural and functional comparative genomic studies on Myrtaceae species.

## 1 Introduction

*Syzygium* is the largest tree genus with about 1,200 species naturally occurring from the Old World tropics and subtropics to the Pacific (POWO, 2023; Craven and Biffin, 2010; Beech et al., 2017). In addition to their ecological importance, the genus includes several species grown for their edible fruit, medicinal properties, timber, and for the horticulture industry (e.g., *S. malaccense, S. aqueum, S. jambos*, and *S. cumini*), the most important economically being the clove tree (*S. aromaticum)* (Parnell et al., 2007; Nurdjannah and Bermawie, 2012; Nair, 2017; Cock and Cheesman, 2018).

The *Syzygium* genus belongs to the Myrtaceae family—the eighth largest family of flowering plants—and includes economically important species such as eucalyptus, myrtle, and guava (Grattapaglia et al., 2012; Christenhusz and Byng, 2016; Saber et al., 2023). Although the majority of species of the Myrtaceae family are diploids (2n = 22) with small to intermediate genome sizes (234–1785 Mb), occasional polyploids derived from the most conserved chromosome number x = 11 were also reported (e.g., within the *Eugenia, Syzygium*, and *Psidium* genera) (Wilson, 2010; Grattapaglia et al., 2012; Tuler et al., 2019; Pellicer and Leitch, 2020; Machado and Forni-Martins, 2022). The *Eucalyptus grandis* genome was released in 2014 as the first reference genome for the Myrtales order and the Myrtaceae family (Myburg et al., 2014). New chromosome-scale assemblies were subsequently published, enabling comparative genomics analyses within the family. Published chromosome-scale genome assemblies for the Myrtaceae currently represent major tribes of the family: Eucalypteae (*Eucalyptus grandis, Corymbia citriodora*, *Eucalyptus urophylla × Eucalyptus grandis*), Leptospermeae (*Leptospermum scoparium*), Myrteae (*Psidium guajava*, *Rhodomyrtus tomentosa*), Metrosidereae (*Metrosideros polymorpha*), Melaleuceae (*Melaleuca alternifolia*), and Syzygieae (*S. aromaticum, Syzygium grande*). These assemblies were generated from diploid specimens, and their size ranged from 297 Mb to 690 Mb (Myburg et al., 2014; Izuno et al., 2019; Thrimawithana et al., 2019; Feng et al., 2021; Healey et al., 2021; Low et al., 2022; Ouadi et al., 2022; Zheng et al., 2022; Li et al., 2023; Shen et al., 2023).

The clove (*S. aromaticum* (L.) Merr. & L.M. Perry) and sea apple (*S. grande*) genomes were constructed using a combination of Oxford Nanopore Technologies long-reads and Illumina short-reads and anchored on 11 chromosomes using Hi-C technologies (Low et al., 2022; Ouadi et al., 2022). The sea apple genome assembly (405 Mb), 182 re-sequenced *Syzygium* species and 58 re-sequenced unidentified taxa were used to generate whole genome-level phylogenies of the *Syzygium* genus, thus providing new insights into the infrageneric classification of *Syzygium*, as well as into the genus diversification patterns and their drivers. The clove genome assembly (370 Mb) was exploited to investigate the genetic basis of the biosynthesis of eugenol, the major biocompound of clove products (Kamatou et al., 2012; Otunola, 2022). To provide insights into the clove genome evolution, comparative genomics analyses were also performed between *S. aromaticum* and *E. grandis*. The synteny analysis performed between these two Myrtaceae species’ genomes assemblies revealed good genome structure conservation. The structures of chromosomes 1, 3, 5, and 7 were found to be highly conserved between *E. grandis* and *S. aromaticum*, and 10 intrachromosomal rearrangements occurring on the 7 other chromosomes were observed (chromosomes 2, 4, 6, 8, 9, 10, and 11). Interestingly, the intrachromosomal rearrangements detected between the two eucalypt species, *E. grandis* and *C. citriodora*, were located on the same seven chromosomes (Butler et al., 2017; Healey et al., 2021). Long terminal repeat retrotransposons (LTR-RTs) are transposable elements (TEs) that move through the genome via a copy-and-paste mechanism using an RNA intermediate. They are considered the most abundant TE component in plant genomes and important drivers of genome size variation and diversification (Wicker et al., 2007; Zhou et al., 2021). Comparing the LTR-RTs repertoires of *S. aromaticum* and *E. grandis* revealed a differential accumulation of the LTR-RTs belonging to the superfamilies Copia and Gypsy between the two species. In *S. aromaticum* genome assembly, the LTR-RTs belonging to the Gypsy superfamily were more abundant than those belonging to the Copia superfamily. In contrast, a higher number of LTR-RTs Copia versus Gypsy was found in the *E. grandis* genome assembly.

No infrageneric comparison of chromosome-scale assemblies has been performed for the *Syzygium* genus. To further investigate the evolution of the genome architecture of *Syzygium* species and verify whether the rearrangements found between *S. aromaticum* and *E. grandis* chromosomes were the consequences of evolutionary events or due to sequencing and assembly artifacts, we generated additional chromosome-scale genome assemblies for *Syzygium malaccense* (L.) Merr. & L.M. Perry, *Syzygium aqueum* (Burm.f.) Alston, *Syzygium jambos* (L.) Alston, and *Syzygium syzygioides* (Miq.) Merr. & L.M. Perry. Like *S. aromaticum* and *S. grande*, the four species belong to the subgenus *Syzygium*, the largest of the five *Syzygium* subgenera for which the crown age was estimated at 9.4 Mya by Low et al. (Low et al., 2022). Previous karyotype studies indicated that *S. malaccense* is a diploid with 2n = 22 chromosomes (Pedrosa et al., 1999) and that *S. jambos* is a tetraploid (2n = 44); however, different chromosome numbers were also reported in the literature for the species (2n = 28, 33, 46, ~54, 66) (Van Lingen, 1991; Oginuma et al., 1993). The chromosomal numbers reported in the literature indicate that *S. aqueum* is also a tetraploid (2n = 44) (Panggabean, 1991).

Here, we describe the *de novo* assembly and annotation for *S. malaccense, S. aqueum*, *S. jambos*, and *S. syzygioides.* To enable subsequent comparative genomic analyses, the four genomes consisting of monoploid consensus (11 chromosomes and unplaced sequences) were generated to achieve the same level of quality for the four species’ genome assemblies and comparable to those of published chromosome-scale assemblies of their Myrtaceae relatives. Then, we compared the genome architecture of the four newly *Syzygium* assembled genomes with those of *S. aromaticum* and *S. grande* and their genome features (gene sets and LTR-RTs repertoires) with those of *S. aromaticum* to investigate genomic evolution from their common ancestors.

## 2 Materials and methods

### 2.1 Biological materials

The *S. malaccense*, *S. aqueum*, *S. jambos*, and *S. syzygioides* genome assemblies were generated from trees growing in the Masoala Hall of the Zurich Zoo in Switzerland. Voucher specimens were deposited in the Zürich herbarium (*S. malaccense* (ZT-00170996), *S. aqueum* (ZT-00170994), *S. jambos* (ZT-00170999), and *S. syzygioides* (ZT-00170991)). Samples collected from the trees were stored at -80°C until nucleic acid extraction.

### 2.2 DNA and RNA isolation

High-molecular-weight genomic DNA was isolated from frozen leaves using the “ONT high-molecular-weight gDNA extraction from plant leaves” protocol (Oxford Nanopore Technologies, Oxford, UK). Following the extraction, we performed a size selection step using the Circulomics Nanobind Plant Nuclei Big DNA Kit from PacBio (Menlo Park, CA, USA). (NB-900-801-001).

Total RNA from *S. malaccense*, *S. aqueum*, *S. jambos*, and *S. syzygioides* were isolated in triplicate from whole leaves (young and mature), lamina of mature leaves, and stems. Total RNA was also isolated in triplicate from *S. syzygioides*’ buds (in the fruiting stage) and *S. jambos*’ buds (before and after flowering) and flowers.

Total RNA was extracted from frozen powder using Ambion PureLink Plant RNA Reagent (Ambion by Life Technologies, Carlsbad, CA, USA). The concentration and quality of the total RNA were assessed with an Agilent Bioanalyzer using the Agilent RNA 6000 Nano Kit (Agilent, Santa Clara, CA, USA).

### 2.3 Illumina sequencing library preparation and sequencing

DNAseq libraries were prepared from total gDNA using the Celero PCR workflow with an enzymatic fragmentation kit from Tecan (Männedorf, Switzerland). DNAseq libraries were loaded on an Illumina S2 flow cell and sequenced on the Illumina Novaseq 6000 instrument (Illumina, San Diego, CA, USA) as 2 x 151 bp paired-end reads.

Hi-C libraries were prepared from 0.2 g of frozen leaves using the Proximo Hi-C Kit following the manufacturer’s instructions (Phase Genomics, Seattle, WA, USA) and sequenced on an Illumina HiSeq 4000 instrument (Illumina) as 2 x 151 bp paired-end reads.

mRNA stranded libraries were prepared from 500 ng of total RNA using the Tecan Universal Plus mRNA-Seq library preparation kit with NuQuant^®^ and sequenced on an Illumina HiSeq 4000 instrument as 2 x 151 bp paired-end reads.

Illumina raw reads generated from DNAseq libraries and Hi-C libraries were cleaned using fastp 0.23.2 (--length_required 75 --low_complexity_filter) (Chen et al., 2018).10.1038/s41597-021-00968-x

### 2.4 ONT sequencing library preparation and sequencing

Sequencing libraries were generated from high-molecular-weight gDNA and prepared for sequencing on PromethION flow cells (FLO-R0002) by using the ligation sequencing (SQK-LSK109) and flow cell priming (EXP-FLP002) kits (Oxford Nanopore Technologies, Oxford, UK). The base calling was performed by using Guppy 6.1.1 and the super accuracy plant model. Raw ONT reads were cleaned using seqkit 2.2.0 (--min-qual 9 --min-len 5000) (Shen et al., 2016) to discard reads shorter than 5,000 bp or with quality scores lower than 9.

### 2.5 Genome profiling

Cleaned Illumina paired-end reads from DNAseq libraries were analyzed by GenomeScope 2.0 and smudgeplot 0.2.4 to estimate the genome size, percentage of heterozygosity, and the ploidy level using a k-mer size equal to 21 (Ranallo-Benavidez et al., 2020).

### 2.6 *De Novo* genome assembly

ONT cleaned reads were corrected with fmlrc2 0.1.7 (--cache_size 13 –K 21 59 79) (Mak et al., 2023) using cleaned Illumina paired-end short-reads from DNAseq libraries. The corrected ONT reads were then assembled using flye 2.9 (--read-error 0.01 --nano-hq) (Kolmogorov et al., 2019) and iteratively polished with ntedit 1.3.5 (-m 2 -i 3 -d 3 -X 0.5 -Y 0.5) using kmer profiles created with nthits 0.0.1 (--solid --outbloom -b 36) for kmers of lengths 60, 50, 40 and 30 (Warren et al., 2019) using Illumina paired-end short reads from DNAseq libraries. Haplotigs were detected and removed from the polished contigs using purge_dups 1.2.5 (Guan et al., 2020) using cutoff of 10, 315 and 645 for *S. malaccense*, 70, 440 and 960 for *S. aqueum*, and 60, 410, 960 for *S. jambos*, 10, 410 and 960 for *S. syzygioides*.

Cleaned Illumina read pairs generated from Hi-C libraries were mapped to the genomes to remove reads with low mapping scores, duplicated reads, and paired-end reads. Illumina Hi-C read pairs were mapped to the haplotig-purged contigs using minimap2 2.24 (Li, 2018) rather than bwa (Li, 2013) since we noticed that it results in assemblies of equivalent qualities in a shorter time. The scaffolding to a chromosome-scale assembly was performed using yahs 1.1a2 (-r 1000,2000,5000,10000,20000,50000,100000,200000,500000,1000000,2000000,5000000) (Zhou et al., 2022). Hi-C map files were generated with PretextMap 0.1.9 (https://github.com/wtsi-hpag/PretextMap) and used to manually curate the assemblies using PretextView 0.2.5 (https://github.com/wtsi-hpag/PretextView).

The curated genome assemblies were mapped to the *S. aromaticum* genome (Ouadi et al., 2022) using minimap2 2.24, visualized using a custom R script, and the orientation and names of the chromosomes were set in accordance with those of *S. aromaticum*. Chromosome-scale assembly completeness was assessed by using the genome evaluation mode of BUSCO 5.4.4 and the eudicots_odb10 lineage dataset (Simão et al., 2015). The QVs of the final assemblies were estimated using yak 0.1 (qv -K 2000000000) with kmer profiles created using yak 0.1 (count -k 31 -K 2000000000 -b37) (Cheng et al., 2021).

### 2.7 Gene annotation

The Illumina RNAseq reads from *S. malaccense*, *S. aqueum*, *S. jambos* and *S. syzygioides* as well as those used for the clove genome annotation were cleaned, and overlapping paired-reads were merged using fastp 0.23.2 (--length_required 75 --low_complexity_filter --merge) (Chen et al., 2018) before being mapped as single cDNA reads to the assemblies using minimap2 2.24 (-ax splice:hq -G5K -N50) (Li, 2018). Gene models were then created for each RNASeq sample using scallop 0.10.5 (--min_transcript_coverage 5 --min_single_exon_coverage 50 --min_splice_bundary_hits 5 --min_mapping_quality 0) (Shao and Kingsford, 2017).This approach was used for the annotation of the clove genome, where it was observed to produce better gene models than by directly mapping paired-reads with a dedicated mapper.

To obtain models for genes that are not expressed in the RNAseq samples, the transcripts from *S. aromaticum* and *E. grandis* gene annotations were mapped to the assemblies using minimap2 2.24 (-ax splice:hq -I5G -G5K -N50 -uf) (Li, 2018), and gene models created using bedtools 2.30.0 (bamtobed -bed12) (Quinlan and Hall, 2010) and custom gawk scripts to convert the obtained bed file into a gtf file.

The final gene models were obtained by merging the RNAseq, *S. aromaticum*, and *E. grandis* gene models using taco 0.7.3 (--gtf-expr-attr TPM --filter-min-expr 10) (Niknafs et al., 2017) and adding coding sequences using Transdecoder 5.5.0 (LongOrfs -S -m 64; Predict --single_best_only --retain_blastp_hits dmd.tsv) (https://github.com/TransDecoder/TransDecoder/wiki), diamond 2.0.15 (blastp --query longest_orfs.pep --db uniref-malvids.dmnd --max-target-seqs 1 --outfmt 6 --evalue 1e-6) (Buchfink et al., 2015) and gffread 0.12.7 (Pertea and Pertea, 2020).

The eudicotyledons portion of UniProt filtered to remove proteins with poor descriptions was used to annotate the gene models with their best hit using diamond 2.0.15 (blastx --query tx.fa --db eudicotyledons.filtered.dmnd --top 10 --min-score 200 --ultra-sensitive --iterate). The illustration of the regions where genes encoding for putative eugenol synthase were predicted was generated using gggenes 0.4.0 (https://github.com/wilkox/gggenes).

### 2.8 Repeat annotation

Annotation of transposable elements was carried out using TE-greedy-nester 1.0.0 (--discovery_tool LTRharvest) (Lexa et al., 2020), genometools LTRharvest 1.6.2 (Ellinghaus et al., 2008) and TEsorter 1.3.0 (-db rexdb-plant --min-coverage 10 --max-evalue 0.01 --pass2-rule 70-30-80) (Zhang et al., 2022) with REXdb (Neumann et al., 2019). The insertion age of the predicted transposable elements was then calculated as previously reported (Marcon et al., 2015). In addition, Red 2.0 (Girgis, 2015), GRF 1.0 (Shi and Liang, 2019) and cd-hit 4.8.1 (grf-main -i genome.fa -c 1 -o genome.MITE --min_tr 10; cd-hit-est -i genome.MITE/candidate.fasta -o genome.MITE/clusteredCandidate.fasta -c 0.90 -n 5 -d 0 -aL 0.99 -s 0.8 -M 0; grf-mite-cluster -i genome.MITE/clusteredCandidate.fasta.clstr -g genome.fa -o genome.MITE) (Fu et al., 2012), EAHelitron (Hu et al., 2019), and tantan 39 (-f4) (Frith, 2011) were used to predict repeats, Miniature Inverted-repeat Transposable Elements (MITEs), helitron, and tandem repeats, respectively.

### 2.9 Synteny analyses

Synteny between the *Syzygium* species was done by pairwise mapping whole genomes using minimap2 2.24 (Li, 2018), identifying structural variants using syri 1.6 (Goel et al., 2019), and plotting syntenic blocks larger than 20 kb using plotsr 0.5.4 (Goel and Schneeberger, 2022).

### 2.10 Orthologue analyses

Orthologous genes were clustered into HOGs with OrthoFinder 2.5.4 (Emms and Kelly, 2019) using the set of predicted protein sequences from the five species assemblies.

## 3 Results

### 3.1 Genome profiling

Smudgeplot and GenomeScope 2.0 were used to perform a genome profiling step using Illumina PE short-reads from DNAseq libraries as input and a K-mer length of 21 bp (Ranallo-Benavidez et al., 2020) (**Table 1**; **Supplementary Table 1**; **Supplementary Figures 1**, **2**). The ploidy level predicted by Smudgeplot was in accordance with previous karyotype studies for the studied *S. malaccense* and *S. jambos* specimens (Oginuma et al., 1993; Pedrosa et al., 1999). *S. malaccense* was predicted to be a diploid specimen (2n = 2x = 22) like *S. aromaticum* and *S. grande*. The *S. aqueum*, *S. jambos*, and *S. syzygioides* specimens were predicted as being autotetraploid (2n = 4x = 44). The estimated monoploid genome sizes were similar among the four *Syzygium* species (343–372 Mb), a size range consistent with the small genome assembly sizes of *S. aromaticum* (370 Mb) and *S. grande* (405 Mb) (Low et al., 2022; Ouadi et al., 2022). The heterozygosity rate estimated by the GenomeScope 2.0 ranged from 2.3% for the diploid specimen *S. malaccense* to 4.3% for the autotetraploid specimen *S. aqueum.* These heterozygosity rates appeared to be higher than for *S. aromaticum* (0.18%) (Ouadi et al., 2022) and the average reported by Ellestad et al., who performed a literature review of the genome-wide heterozygosity values estimated using the software GenomeScope and GenomeScope 2.0 (Ellestad et al., 2022). They found that the average value inferred for all plant species assessed was 1.59% (1.10% for diploid plants only) noting that over half of the plant species considered were cultivated for human usage, which could affect the average value accuracy.

**Table 1:** Genome profiling summary.

	*S. aromaticum* (Ouadi et al., 2022)	*S. malaccense*	*S. aqueum*	*S. jambos*	*S. syzygioides*
Predicted ploidy	2n = 2x = 22	2n = 2x = 22	2n = 4x = 44	2n = 4x = 44	2n = 4x = 44
Estimated genome (1x) size	343 Mb	372 Mb	345 Mb	361 Mb	372 Mb
Estimated heterozygosity rate	0.18%	2.30%	4.30%	3.60%	4.10%

### 3.2 Genome *De Novo* assembly

The four *de novo* chromosome-scale assemblies were constructed using long-reads from Oxford Nanopore Technologies (ONT), short paired-end reads from Illumina DNAseq libraries, and Hi-C libraries generated for each *Syzygium* species (**Supplementary Tables 1, 2**).

**Figure 1:**
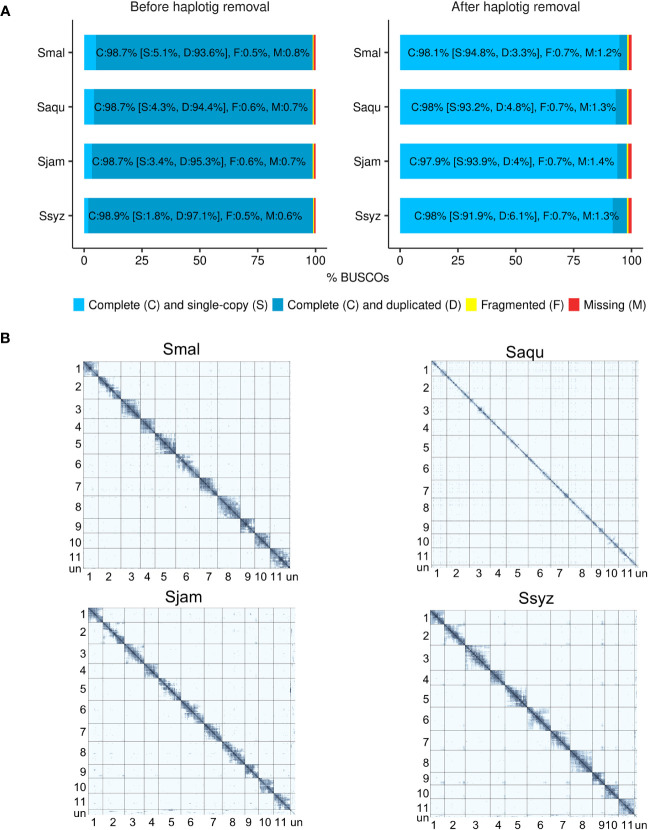
Assessment of the efficiency of the haplotig removal step and Hi-C scaffolding. **(A)** BUSCO completeness score comparison of the polished contigs before and after the haplotig removal step for *S. malaccense* (Smal), *S. aqueum* (Saqu), *S. jambos* (Sjam), and *S. syzygioides* (Ssyz) (BUSCO version 5.4.4 - dataset: eudicots_odb10 (n = 2326)). **(B)** Hi-C contact maps showing the Hi-C interactions among the 11 assembled chromosomes and unplaced scaffolds (un) for each species.

To prevent assembly artifacts possibly caused by heterozygosity and polyploidy of the *Syzygium* specimens, haplotigs were detected and removed from the polished contigs. The effect of the haplotig removal step was assessed using BUSCO (Benchmarking Universal Single-Copy Orthologs) in genome mode (Simão et al., 2015). After the haplotig removal step, the number of complete and duplicated BUSCOs genes was considerably reduced in the haplotig-purged contigs (3.3% to 6.1%) when compared to the polished contigs (93.6% to 97.1%) (**Figure 1A**). Hi-C data enabled the scaffolding of contigs into 11 chromosomes. On the Hi-C contact matrices, a strong intra-chromosomal signal indicates efficient scaffolding, with the 11 chromosomes of each *Syzygium* assembly supported by a high number of their respective Hi-C reads (**Figure 1B**).

**Figure 2:**
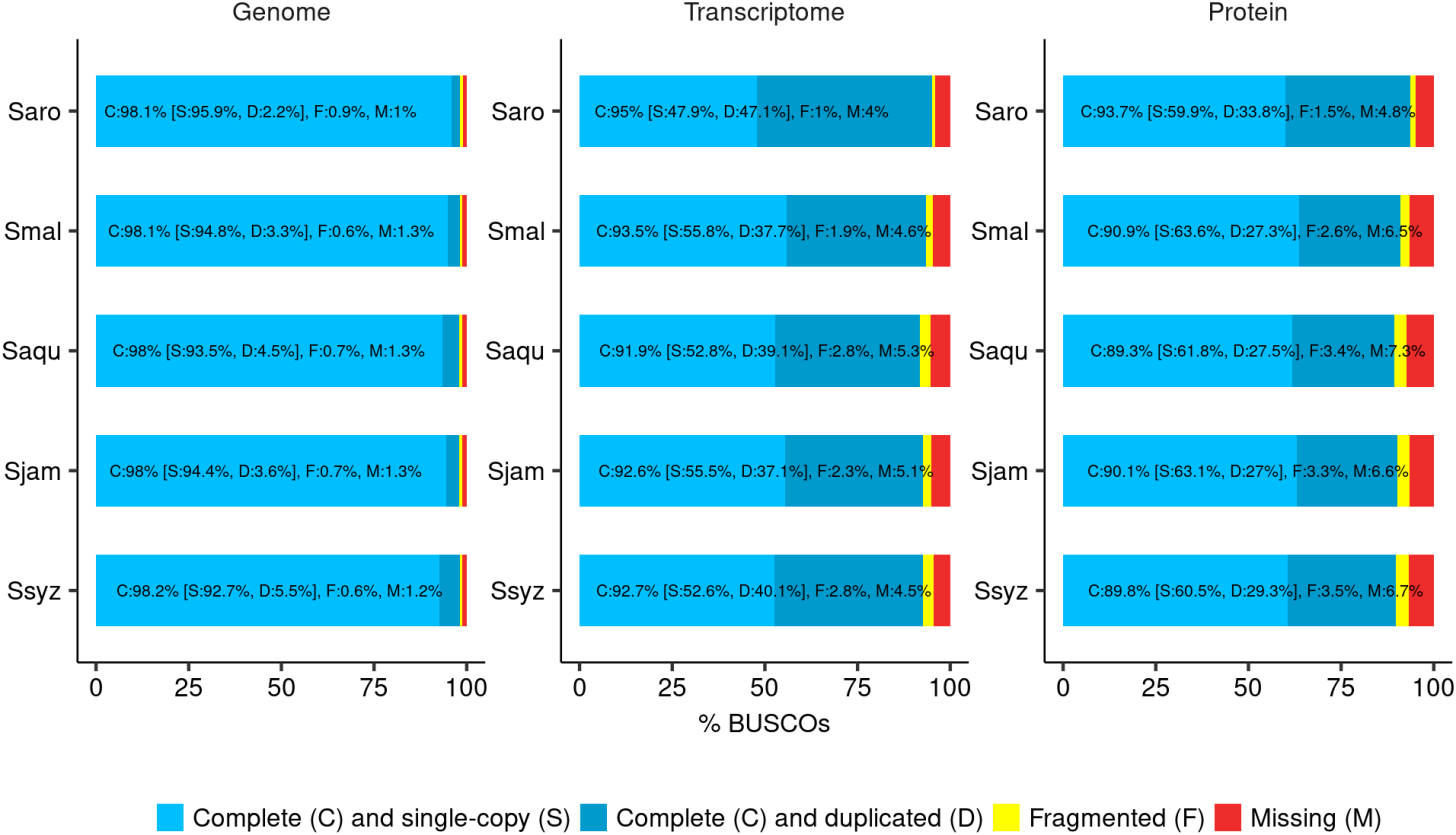
BUSCO completeness assessment. Assessment of the final genome assembly, transcript set, and protein set of *S. aromaticum* (Saro), *S. malaccense* (Smal), *S. aqueum* (Saqu), *S. jambos* (Sjam), and *S. syzygioides* (Ssyz) (BUSCO version 5.4.4 - dataset: eudicots_odb10 (n = 2326)).

The final chromosome-scale assemblies for *S. malaccense* (430 Mb), *S. aqueum* (392 Mb), *S. jambos* (426 Mb), and *S. syzygioides* (431 Mb) consisted of monoploid consensus (11 chromosomes and unplaced sequences) with comparable quality metrics. A high level of quality at the base-scale (quality value [QV] between 44.006 and 45.114), of contiguity (97.5% to 99.8% of the assemblies length anchored on 11 chromosomes) and completeness (BUSCO complete genes scores of 98%) was reached for the four new assembled *Syzygium* genomes (**Table 2**; **Figure 2**; **Supplementary Tables 3, 4**).

**Table 2:** Assembly and annotation statistics.

	*S. malaccense*	*S. aqueum*	*S. jambos*	*S. syzygioides*
Assembly				
Number of scaffolds	23	54	117	101
Number of chromosome-scale scaffolds	11	11	11	11
Proportion of undetermined bases (N)	0.01%	0.01%	0.02%	0.02%
QV^1^ of the assembly	45.114	44.006	44.028	44.292
Length of assembly (bp)	429,836,287	391,897,832	426,159,599	431,079,378
Length of chromosome-scale scaffolds (bp)	429,008,219	386,536,673	415,622,982	424,827,227
Gene annotation				
Number of predicted genes	30,842	29,879	31,611	32,142
Number of predicted transcripts	57,144	55,010	57,897	59,495
Average transcript length (bp)	2010.89	2008.09	1991.19	2007.31
Average CDS^2^ length (bp)	1122.42	1124.09	1116.93	1100.23
Average exon per transcript	5.62	5.67	5.59	5.66
Repeat annotation				
Repeat sequences (bp)	184,916,857 (43.02%)	162,020,435 (41.34%)	180,563,593 (42.37%)	184,003,101 (42.68%)
LTR^3^ retrotransposons (bp)	96,086,564 (22.35%)	74,914,968 (19.12%)	77,407,268 (18.16%)	73,171,928 (16.97%)
LTR Gypsy (bp)	62,668,430 (14.58%)	48,141,612 (12.28%)	45,090,450 (10.58%)	40,369,474 (9.36%)
LTR Copia (bp)	31,769,467 (7.39%)	25,148,956 (6.42%)	30,040,740 (7.05%)	30,532,813 (7.08%)

^1^ QV, Quality value.

^2^CDS, Coding sequence.

^3^LTR, Long Terminal Repeat.

Despite their high heterozygosity rate, the quality metrics for the genome assemblies of the diploid specimen *S. malaccense* and the autotetraploids *S. aqueum*, *S. jambos*, and *S. syzygioides* were comparable to those reported for *S. aromaticum* assembly (370 Mb) (Ouadi et al., 2022). Nevertheless, BUSCO scores revealed a higher percentage of complete and duplicated BUSCOs in the four new assemblies compared to *S*. *aromaticum* (2.2%), principally in the genome assembly of the three autotetraploid specimens (3.3% to 5.5%) (**Figure 2**).

### 3.3 Genome annotation

The average number of protein-coding genes predicted for the four newly assembled genomes is 31,119, representing 26.52% of the genome assemblies’ size (**Table 2**).

The annotation completeness was assessed using the BUSCO method in transcriptome and protein modes and by selecting the whole set of predicted transcripts and proteins for each gene as inputs, respectively (**Figure 2**; **Supplementary Table 3**). BUSCO results indicated that the annotation completeness is comparable among the four newly assembled *Syzygium* species, with complete BUSCO scores ranging from 91.9% in *S. aqueum* assembly to 93.5% in *S. malaccense* assembly in transcript mode and from 89.3% in *S. aqueum* assembly to 90.9% in *S. malaccense* assembly in protein mode. BUSCO scores obtained for *S. aromaticum* by using the same assessment methods (95% in transcriptome mode and 93.7% in protein mode) were slightly superior to those of newly assembled genomes but still comparable. The loss of complete BUSCOs between the genome and protein mode assessments ranged from 7.2% in *S. malaccense* assembly to 8.7% in *S. aqueum* assembly, indicating acceptable quality of the predicted gene models and protein sets.

**Figure 3:**
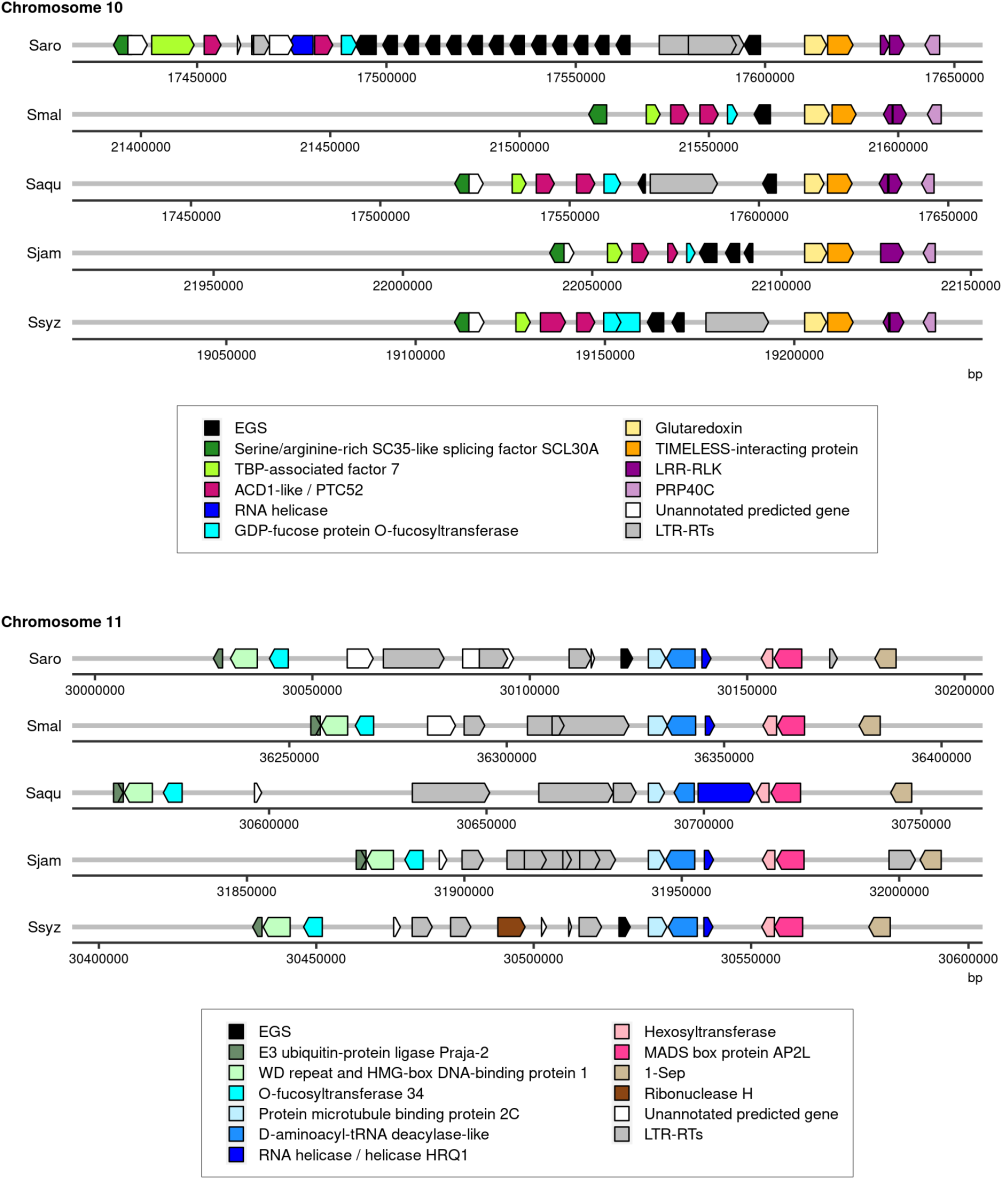
Illustration of the regions of chromosomes 10 and 11 of *S. aromaticum* (Saro), *S. malaccense* (Smal), *S. aqueum* (Saqu), *S. jambos* (Sjam), and *S. syzygioides* (Ssyz) where genes encoding for EGS were predicted. The position (bp) and orientation of the predicted genes on the chromosomes are indicated by arrows colored according to the functional annotation. EGS, accelerated cell death (ACD1), Protochlorophyllide-dependent translocon component Tic52 (PTC52), leucine-rich repeat receptor-like protein kinase (LRR-RLK), Pre-mRNA-processing protein 40C-like (PRP40C), TATA-binding protein-associated factor 7 (TBP-associated factor 7), LTR-RTs.

The genome assembly of *S. aromaticum* comprised multiple copies of a gene encoding for putative eugenol synthase (EGS), the enzyme that catalyzes the synthesis of eugenol from coniferyl acetate. In total, 15 copies split into 2 loci were reported: a first locus on chromosome 10 comprising 14 copies and a second locus on chromosome 11 with 1 copy (Ouadi et al., 2022). The functional annotation of the four newly assembled *Syzygium* species genomes revealed fewer genes encoding for putative EGS. One gene encoding for putative EGS was identified in the genome assembly of *S. malaccense*, two in the genome assembly of *S. aqueum*, and three copies were found in the genome assemblies of *S. jambos* and *S. syzygioides*. All putative EGS genes were located on chromosome 10 except for one of the three copies of *S. syzygioides* located on chromosome 11 (**Figure 3**; **Supplementary Table 5**).

Effective lengths of repeat elements, which are different from their genomic length, were calculated by removing the length of the nested elements they contained. The proportions of genome assembly length occupied by predicted genes (25.97% to 27.37%) and repeat sequences (41.34% to 43.02%) appear to be conserved among the four newly sequenced *Syzygium* genomes (**Table 2**). Using the same method, repeat elements in *Syzygium aromaticum* genome assembly represents 39.98%. The most abundant repeat elements identified in the four newly sequenced *Syzygium* genomes were the LTR-RTs spanning 16.97% of the assembly length for *S. syzygioides* to 22.35% for *S. malaccense*. As reported for *S. aromaticum* and *S. grande*, LTR-RTs belonging to the Gypsy superfamily were more abundant than elements belonging to the Copia superfamily in the four newly sequenced genomes (**Table 2**; **Supplementary Tables 6–9**) (Low et al., 2022; Ouadi et al., 2022).

### 3.4 Synteny analyses

**Figure 4:**
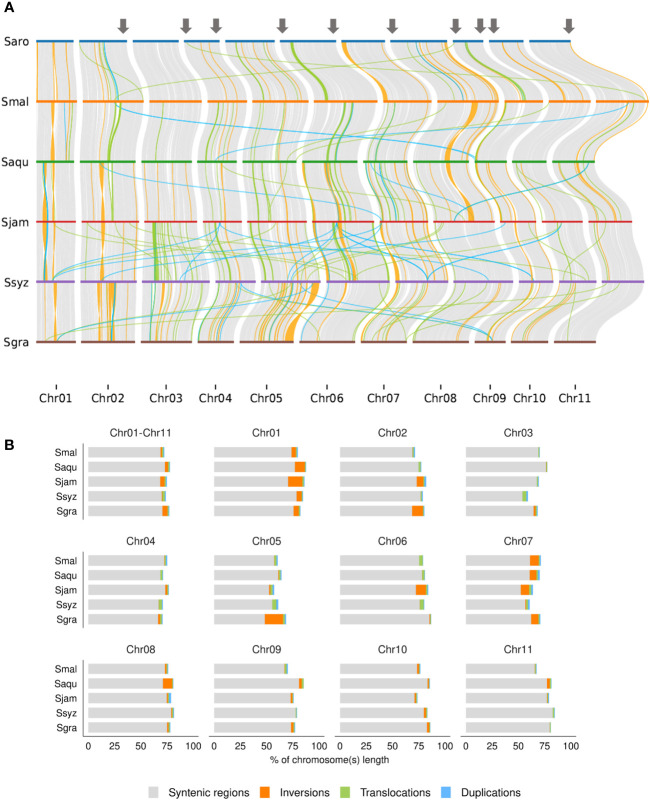
Identification of syntenic and rearranged regions between the 11 chromosomes of *S. aromaticum* (Saro), *S. malaccense* (Smal), *S. aqueum* (Saqu), *S. jambos* (Sjam), *S. syzygioides* (Ssyz), and *S. grande* (Sgra). **(A)** Representation of the alignment of the chromosomal DNA sequences showing syntenic regions, interchromosomal, and intrachromosomal rearrangements larger than 20 kb (inversions, translocations, and duplications). Grey arrows indicate regions where rearrangements were reported between chromosomes of *E grandis* and *S. aromaticum*. **(B)** Pairwise comparison of the percentage of chromosome length occupied by syntenic regions and rearrangements between the chromosome-scale assembly (Chr01-Chr11) and 11 chromosomes (Chr01 to Chr11) of *S. aromaticum* with those of *S. malaccense*, *S. aqueum*, *S. jambos*, *S. syzygioides*, and *S. grande*.

To identify evolutionary structural changes among the *Syzygium* species chromosomes, we performed a synteny analysis on the four newly assembled genomes, *S. aromaticum* and *S. grande*. The alignment of the 11 chromosomes’ DNA sequences of the 6 *Syzygium* species revealed a high conservation of the chromosomal organization (**Figure 4A**).

No large interchromosomal rearrangements were detected between the chromosomes of the six *Syzygium* species. A high percentage of the five species’ chromosome lengths were syntenic with *S. aromaticum*, ranging from 68.45% between *S. aromaticum* and *S. jambos* to 73.02% between *S. aromaticum* and *S. aqueum*. Intrachromosomal rearrangements such as inversions, translocations, and duplications between the chromosomes of *S. aromaticum* and those of the other five *Syzygium* species represented 5% of their 11 chromosomes length on average. In terms of number, the most frequent rearrangements observed between *S. aromaticum* and the five other species were duplications and translocations with average numbers of 1348 and 1325, respectively, spanning an average of 0.85% to 1.43% of the 11 chromosome lengths. Inversions were found less frequently for all species but occupied a larger fraction of the genome assemblies’ length than duplications and translocations except for *S. syzygioides*. The percentage of assembly lengths comprising inversions between *S. aromaticum* and the five other *Syzygium* species ranged from 0.68% between *S. aromaticum* and *S. syzygioides* to 4.83% between *S. aromaticum* and *S. grande*. Overall, the size of the inversions was relatively small. For instance, 11 inversions were detected, between chromosome 5 of *S. aromaticum* and *S. grande*, representing 17.32% of the chromosome length of *S. grande* (41,797,999 bp) and 1.87% of its 11 chromosomes (387,620,547 bp) (**Figure 4B**; **Supplementary Table 4**). In contrast, the synteny analysis performed between *S. aromaticum* and *E. grandis* revealed 10 intrachromosomal rearrangements on chromosomes 2, 4, 6, 8, 9, and 10 that included large terminal inversions representing up to 40% of the chromosome length of *S. aromaticum*. The other four chromosomes (1, 3, 5, and 7) of the two Myrtaceae species were highly syntenic (Ouadi et al., 2022). To further investigate the chromosomal architecture evolution of the *Syzygium* species and verify that these rearrangements were due to biological events rather than assembly artifacts, we also performed DNA alignment of the chromosome sequences of *E. grandis* with the those of *S. malaccense*, *S. aqueum*, *S. jambos*, and *S. syzygioides*. Chromosomes 1, 3, 5, and 7 of *E. grandis* and those of the four newly assembled species were also highly syntenic, and we observed the same 10 rearrangements on chromosomes 2, 4, 6, 8, 9, 10 and 11 (**Figure 4A**; **Supplementary Figure 3**).

### 3.5 Gene orthology

To investigate the phylogenetic relationships among gene sequences of *S. aromaticum*, *S. malaccense*, *S. aqueum*, *S. jambos*, and *S. syzygioide*s, the sets of predicted protein sequences from the five species assemblies were analyzed using OrthoFinder (Emms and Kelly, 2019).

**Figure 5:**
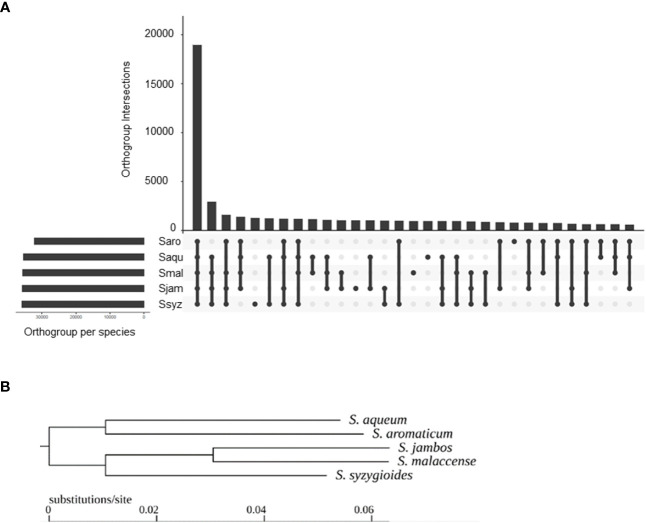
Hierarchical orthogroups (HOGs) inferred by OrthoFinder between S*. aromaticum* (Saro)*, S. malaccense* (Smal), *S. aqueum* (Saqu), *S. jambos* (Sjam), and *S. syzygioides* (Ssyz). **(A)** Number of HOGs inferred by OrthoFinder using the set of predicted proteins for the five *Syzygium* species. **(B)** Rooted species tree inferred by OrthoFinder.

A total of 49,269 hierarchical orthogroups (HOGs) were identified, including 93.7 to 95.2% of each species gene set (**Figure 5A**). Of these, 18,963 (38.5%) HOGs contained genes from all five species, and 4,928 (10%) were species specific. In more detail, 789 were specific to *S. aromaticum*, 950 were specific to *S. malaccense*, 940 HOGs were specific to *S. aqueum*, 1009 HOGs were specific to *S. jambos*, and 1240 HOGs were specific to *S. syzygioides*. Pairwise, *S. aromaticum* and *S. aqueum* appear to share the lowest number of orthogroups (625). The highest number of shared HOGs inferred between each pair of studied species was found between *S. aqueum* and *S. syzygioides* (1218), followed by *S. aqueum* and *S. malaccense* (1152), and *S. jambos* and *S. malaccense* (1027). The species tree resulting from the analysis of the HOGs divided the *Syzygium* species studied into two groups based on closer relationships: the first group comprising *S. aromaticum* and *S. aqueum* and a second group comprising *S. jambos*, *S. malaccense*, *and S. syzygioides* (**Figure 5B**).

### 3.6 Annotation and comparison of LTR-RTs Gypsy and Copia repertoires

To clarify the dynamic activity of full-length LTR-RTs belonging to the superfamilies Gypsy and Copia within the *Syzygium* genus, we identified the lineages belonging to each superfamily located on the chromosomes of *S. malaccense* (429 Mbp), *S. aqueum* (387 Mbp), *S. jambos* (416 Mbp), and *S. syzygioides* (425 Mbp) and estimated their insertion time. Then, we compared the repertoires’ compositions and repeat element insertion times of the four species with those of *S. aromaticum* (368 Mbp).

**Figure 6:**
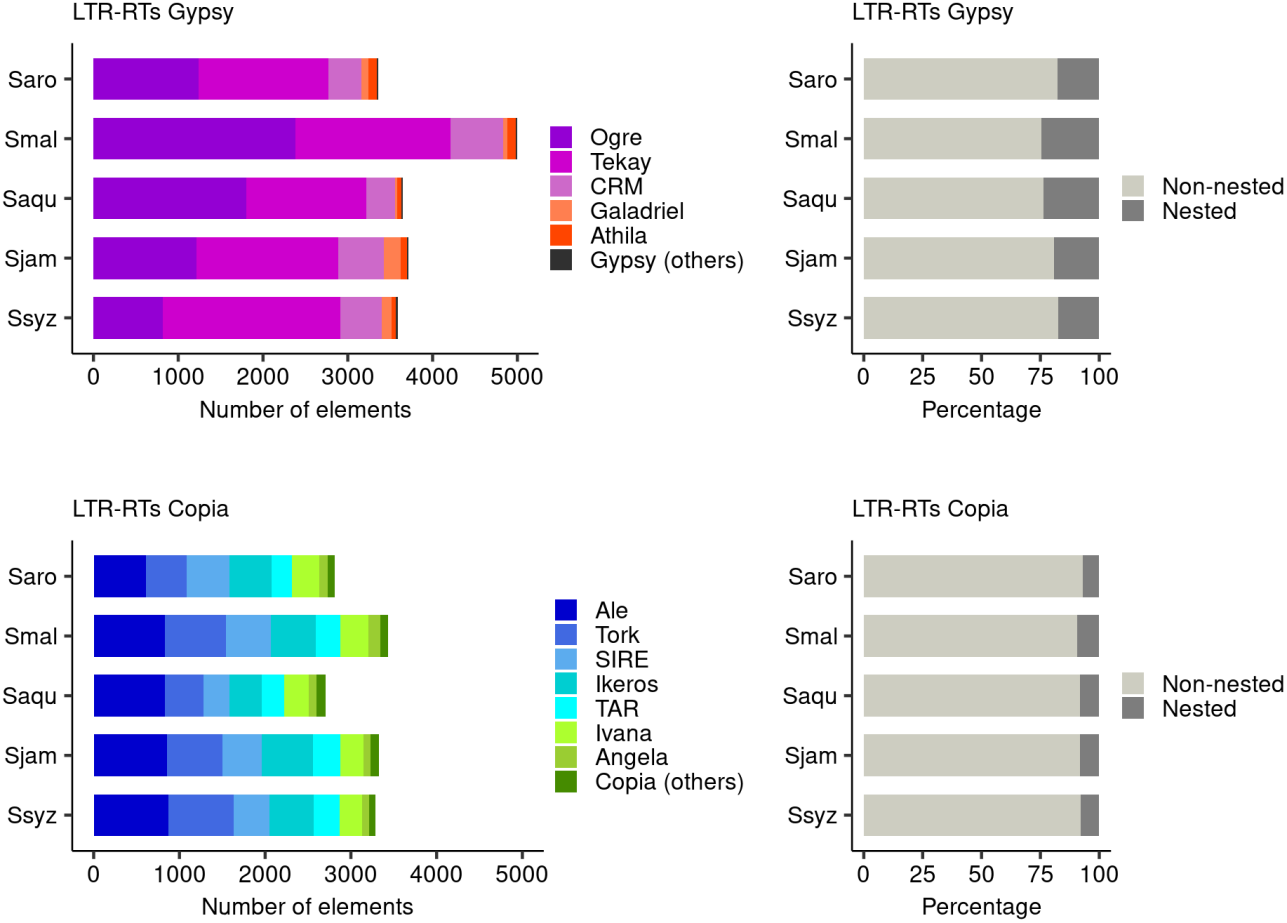
Composition of the full-length LTR-RTs Gypsy and Copia repertoires. **(A)** Number of elements belonging to the Gypsy and Copia lineages identified on the 11 chromosomes of *S. aromaticum* (Saro), *S. malaccense* (Smal), *S. aqueum* (Saqu), *S. jambos* (Sjam), and *S. syzygioides* (Ssyz). **(B)** Proportion of nested and non-nested elements. Gypsy (others) group comprises the lineages non-chromo-outgroup, Reina, Retand, tatIII, and elements Gypsy to which no lineages were assigned. Copia (others) group comprises the lineages Alesia, Bianca, Gymco-I, Gymco-IV, Gymco-II, and Osser.

We found that *S. malaccense* and *S. aromaticum*, the largest and smallest chromosome-scale assemblies of this study, contained the highest (8427) and lowest number (6167) of LTR-RTs in Gypsy and Copia, respectively (**Figure 6A**; **Supplementary Tables 6–9**). In the five *Syzygium* species’ chromosomes, we identified a higher number of LTR-RTs for Gypsy than Copia, with a ratio of Gypsy to Copia content ranging from 1.09 for *S. syzygioides* to 1.45 for *S. malaccense*. The Gypsy superfamily comprised a higher proportion of nested elements (17.37% to 24.47%) compared to the Copia superfamily (7.01% to 9.44%), suggesting distinct accumulation and mobile activity of both superfamilies in all five species. Our results revealed little variation in the number of Copia elements on the chromosomes of *S. aqueum* (2705 elements) and *S. aromaticum* (2809), the two smallest chromosome-scale assemblies, and on the chromosomes of *S. syzygioides* (3290), *S. jambos* (3324), and *S. malaccense* (3433). In contrast, we found a notably higher accumulation of Gypsy elements (4994) in the chromosomes of *S. malaccense* compared to the four other species. The ratio of Gypsy content varied from 1.35 when comparing *S. malaccense* with *S. jambos* to 1.49 when comparing *S. malaccense* with *S. aromaticum*. It represented a difference in Gypsy effective length of 19,402,234 bp to 21,766,176bp, respectively. In the five *Syzygium* chromosome-scale assemblies, the most abundant lineage belonged to the Gypsy superfamily, but it varied according to the species. The Gypsy lineage Tekay was the most represented for *S. aromaticum* (1534 elements), *S. jambos* (1674 elements), and *S. syzygioides* (2090 elements). At the same time, for S. *malaccense* and *S. aqueum*, we found a higher abundance of the gypsy lineage Ogre (2382 and 1799 elements, respectively). Among the Gypsy superfamily, the most abundant lineages, Tekay and Ogre, were those with the highest proportion of nested elements (19.10% to 28.55% and 16.69% to 27.92%, respectively) in all five species. For *S. aromaticum*, *S. malaccense*, and *S. syzygioides*, the proportion of nested elements belonging to the Athila lineage was also among the highest identified (**Figure 6B**; **Supplementary Figures 4, 5**).

Regarding the Copia superfamily, the most represented lineages on the chromosomes of the five *Syzygium* species were Ale (608 to 873 elements), followed by the lineage Tork (456 to 762 elements) for *S. malaccense*, *S. aqueum, S. jambos* and *S. Syzygoides*, and the lineage SIRE (502 elements) for *S. aromaticum*.

**Figure 7:**
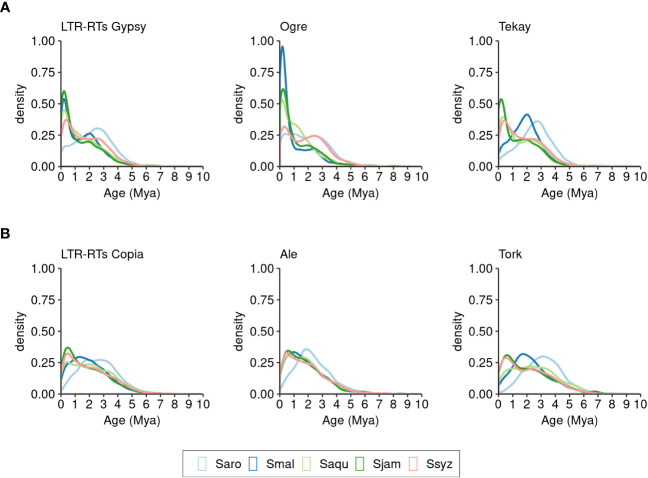
Distribution of insertion times of full-length LTR-RTs of *S. aromaticum* (Saro), *S. malaccense* (Smal), *S. aqueum* (Saqu), *S. jambos* (Sjam), and *S. syzygioides* (Ssyz). **(A)** LTR-RTs Gypsy. **(B)** LTR-RTs Copia.

The insertion times of 97.13% of the full-length Gypsy and Copia elements identified in the five *Syzygium* species were estimated (33,861elements). Nearly all elements (97.33%) were inserted in the last 5 million years (32,958 elements) (**Figure 7**). During this time period, distinct insertion activities of the two superfamilies occurred in the five *Syzygium* species.

Compared to the other four *Syzygium* species, the chromosomes of *S. aromaticum* underwent a more ancient wave of Gypsy insertions (peak at ~2.5 million years ago [Mya]), principally attributed to the Tekay elements, the most abundant lineage in this species (**Figure 7A**). We also found that a few recent insertions (18.02% of insertions) occurred in *S. aromaticum* chromosomes within the last one million years. In contrast, a recent burst of Gypsy insertions (~0–1 Mya) occurred in four other species chromosomes: most insertions of Gypsy in *S. malaccense* (44.53%), *S. aqueum* (44.43%), *S. jambos* (52.55%), and *S. syzygioides* (36.45%) were less than one million years old. We inferred that the high number of Gypsy LTR-RTs found in *S. malaccense* may be attributable to two successive waves of insertions: a peak of Tekay insertions at ~2 Mya and a more recent peak of Ogre at ~1 Mya.

Similar to what we observed for the Gypsy superfamily, the insertion of Copia elements occurred earlier in *S. aromaticum* compared to the four other species, with fewer recent insertions (**Figure 7B**). Compared to the Gypsy elements, a smaller proportion of recent Copia insertions (less than one million years old) were detected in *S. aromaticum* (10.66%), *S. malaccense* (24.16%), *S. aqueum* (26.49%), and *S. jambos* (36.90%) suggesting a distinct recent insertion pattern of the two superfamilies in the four species. However, we found a comparable proportion of Gypsy (36.45%) and Copia (32.22%) elements that were less than one million years old in *S. syzygioides*, the species for which we found the lowest ratio of Gypsy to Copia content (1.09).

## 4 Discussion

Plant genome size, ploidy level, and heterozygosity rates are challenges for genome assembly and annotation. However, lower sequencing costs and recent advances in long-read sequencing technologies, Hi-C technologies, and bioinformatics tools have facilitated the generation of assemblies with high contiguity up to the chromosome-scale also for non-model plants or non-major plant crops (Kyriakidou et al., 2018; Pucker et al., 2022). Newly assembled and annotated genomes from related species can then be used to perform comparative genomics analyses to investigate plant genome evolution and function. Third-generation long-reads from Oxford Nanopore Technologies and Illumina short-reads combined with the Hi-C technology enabled the *de novo* assembly of the chromosome-scale genome for *S. malaccense, S. aqueum*, *S. jambos*, and *S. syzygioides*. A high level of quality at the base level, contiguity, and completeness was reached for the four newly sequenced genomes. The quality of the newly assembled *Syzygium* species genomes were comparable to that of the *S. aromaticum* genome. The slight differences found between the species assemblies’ quality metrics may be linked to the combined impact of the ploidy level and high heterozygosity rates of the four newly sequenced species on the assembly process.

Previous infrageneric comparative genetic mapping analyses revealed high levels of synteny and collinearity among the *Eucalyptus* genus (Hudson et al., 2012; Li et al., 2015). In addition, genomic synteny analyses conducted between the *de novo* assembly of *E. urophylla* × *E. grandis* (EUC) and 30 *Eucalyptus* species revealed that the genome structure of EUC, *E. grandis*, and *E. globulus* showed the higher collinearity, and the absence of large-scale structural variation. Nevertheless, large structural variations among the different chromosomes of the EUC and other *Eucalyptus* species were also detected (Shen et al., 2023). We found that the six *Syzygium* genomes studied were highly syntenic. The intrachromosomal rearrangements (duplications, translocations, and inversions) observed between *S. aromaticum* and the five other *Syzygium* species represent a small percentage (~5% on average) of the 11 chromosomes’ length. These intrachromosomal rearrangements could result from contigs that were not well placed because of Hi-C signals that were not strong enough to correctly determine their position and orientation; however, they may also result from the six species’ distinct genome evolutions.

Organizational conservation of chromosomes 2, 4, 6, 8, 9, 10, and 11 among the six *Syzygium* species studied constitutes new evidence supporting the 10 intrachromosomal rearrangements previously reported on these chromosomes between *S. aromaticum* and *E. grandis* genomes (Ouadi et al., 2022). These 10 rearrangements were also observed when aligning the DNA sequences of the chromosomes of *E. grandis* with those of *S. malaccense*, *S. aqueum*, *S. jambos*, and *S. syzygioides.* Among the rearrangements reported between the chromosomes of *S. aromaticum* and *E. grandis*, similar large terminal inversions on chromosomes 4, 9, 10, and 11 were also reported in the two eucalypts *E. grandis* and *C. citriodora* suggesting that these terminal inversions occurred on *E. grandis* chromosomes (Butler et al., 2017). Two other large terminal inversions were detected between chromosomes 4 and 9 of *S. aromaticum* and *E. grandis* but not between *C. citriodora* and *E. grandis*. These inversions were also observed when comparing the chromosome sequences of *E. grandis* with those of the four newly assembled genomes, suggesting that these inversions resulted from an evolution of the chromosome organization rather than from sequencing and assembly artifacts. Further comparative genomics analyses will be needed with additional *Syzygium* and Myrtaceous species to determine if these inversions are specific to the *Syzygium* genus or subgenus, for which the crown ages were estimated at 51.2 Mya and 9.4 Mya, respectively, (Low et al., 2022).

The analyses of the phylogenetic relationships between gene sequences of *S. aromaticum*, *S. malaccense*, *S. aqueum*, *S. jambos*, and *S. syzygioides* and comparisons of their full-length LTR-RTs repertoires provided insights into the distinct genome evolution of each species following the divergence of the *Syzygium* subg. *Syzygium* species 9.4 Mya (Low et al., 2022). The species tree inferred by OrthoFinder indicated that pairwise *S. aromaticum* and *S. aqueum* and *S. malaccense* and *S. jambos* were closely related, which is consistent with the genome-level phylogenetic trees generated by Low et al. (Low et al., 2022). We observed older waves of LTR-RTs Gypsy and Copia insertions in *S. aromaticum* and fewer insertions less than 1 million years old in the *S. aromaticum* chromosomes compared to those of the four other species studied. In plants, the RNA Directed DNA Methylation (RdDM) pathway, a *de novo* DNA methylation mechanism involving small interfering RNA, plays an important role in TE repression (Wambui Mbichi et al., 2020). Further detailed analysis such as DNA methylation studies will be valuable to clarify the molecular causes of the recent low insertion number of LTR-RTs elements observed in *S. aromaticum*.

*S. aromaticum* is cultivated to produce clove bud (the dried, unopened flower bud), essential oil (EO), and oleoresins rich in eugenol (Nurdjannah and Bermawie, 2012). The EO of *S. aromaticum* contains ~72 to 96.6% of eugenol, while the EO of *S. aqueum* has 0.19% eugenol (Razafimamonjison et al., 2014; Sobeh et al., 2016). Eugenol is a phenylpropane with multiple pharmaceutical activities and is considered a promising alternative drug for human health (e.g., cancer and pathogenic microorganism resistance, diabetes, obesity, and autoimmune diseases) (Kamatou et al., 2012; Batiha et al., 2020; Otunola, 2022). The genome assembly of *S. aromaticum* was exploited to investigate the genetic basis of this important characteristic. The identification of gene families involved in eugenol biosynthesis revealed the presence of multiple copies of genes encoding EGS, which catalyzes the synthesis of eugenol from coniferyl acetate. A cluster of 14 copies was reported on chromosome 10, and additional copies were located on chromosome 11 of *S. aromaticum*. In the genome assembly of the four newly sequenced species, we found fewer gene copies on chromosome 10 (1 to 3 copies) and no copies on chromosome 11 of *S. malaccense*, *S. aqueum*, and *S. jambos*. The presence of this structural variation suggested that a gene-dosage effect may be associated with the high amount of eugenol. Further studies are needed to elucidate the biological functions of the EGS gene copies in *S. aromaticum* and the four other species (e.g., *in vitro* characterization).

*S. malaccense*, *S. aqueum*, and *S. jambos* are grown for their edible fruit. Like *S. aromaticum* and other *Syzygium* species, they are also used in traditional medicine. Research on their numerous pharmaceutical properties has been undertaken (e.g., analgesic, anti-inflammatory, antioxidant, hepatoprotective, antidiabetic, antifungal, antibacterial, antiviral, and anticancer activities) (Nair, 2017; Cock and Cheesman, 2018). For instance, *S. jambos* is traditionally used to treat hemorrhages, wounds, and ulcers; *S. malaccense* is used to treat mouth ulcers and diabetes; and *S. aqueum* to treat diabetes and childbirth pain (Uddin et al., 2022). The chromosome-scale assemblies for these species are new valuable resources for the Myrtaceae family. Combined with other comparative genomics and multi-omics studies, they can be used to further investigate the genomic evolution of the Myrtaceous species and to study the genetic basis of important agronomical traits and biosynthesis of secondary metabolites.

## Data availability statement

The datasets presented in this study can be found in online repositories. The names of the repository/repositories and accession number(s) can be found below: https://www.ncbi.nlm.nih.gov/, PRJNA962868 https://www.ncbi.nlm.nih.gov/, PRJNA962711 https://www.ncbi.nlm.nih.gov/, PRJNA962713 https://www.ncbi.nlm.nih.gov/, PRJNA962712 https://www.ncbi.nlm.nih.gov/genbank/, JASUUE000000000 https://www.ncbi.nlm.nih.gov/genbank/, JASUUB000000000 https://www.ncbi.nlm.nih.gov/genbank/, JASUUC000000000 https://www.ncbi.nlm.nih.gov/genbank/, JASUUD000000000 https://zenodo.org/, 7870328 https://zenodo.org/, 7870326 https://zenodo.org/, 7870330 https://zenodo.org/, 7870334.

## Author contributions

SO performed the laboratory work, analyzed data, and wrote the manuscript. NS performed computational analysis of sequencing data, conceived, and supervised the study, and contributed to manuscript writing. FK, and NI conceived and supervised the study and contributed to manuscript writing. All authors contributed to the article and approved the submitted version.
