# Supplementary material for: Chromosome-scale assemblies of S. malaccense, S. aqueum, S. jambos, and S. syzygioides provide insights into the evolution of Syzygium genomes
Source: Front Plant Sci. 2023 Oct 6;14:1248780. doi: 10.3389/fpls.2023.1248780 (PMC10587690; doi:10.3389/fpls.2023.1248780)
Supplement: Supplementary file 1 [file DataSheet_1.docx]

Supplementary Material

**Chromosome-scale assemblies of *S. malaccense, S. aqueum, S. jambos,* and *S. syzygioides* provide insights into the evolution of *Syzygium* *g*enomes**

**Sonia Ouadi, Nicolas Sierro, Felix Kessler, Nikolai V. Ivanov^*^**

***** **Correspondence:** Nikolai V. Ivanov: nikolai.ivanov@unine.ch

**
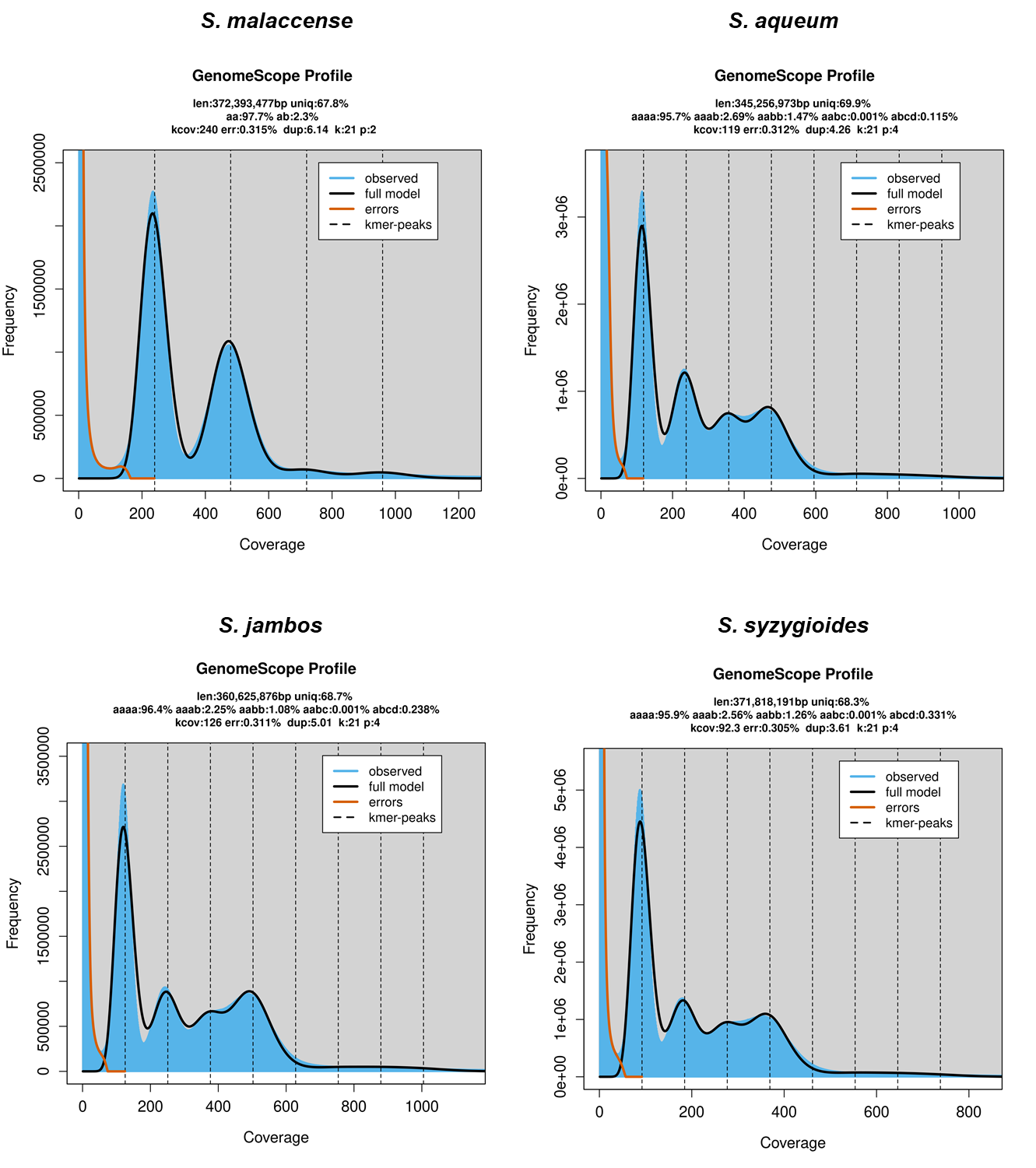
**

**Supplemental Figure 1.** GenomeScope 2.0 profile (k-mer = 21 bp) for the *S. malaccense*, *S. aqueum*, *S. jambos*, and *S. syzygioides* genomes.


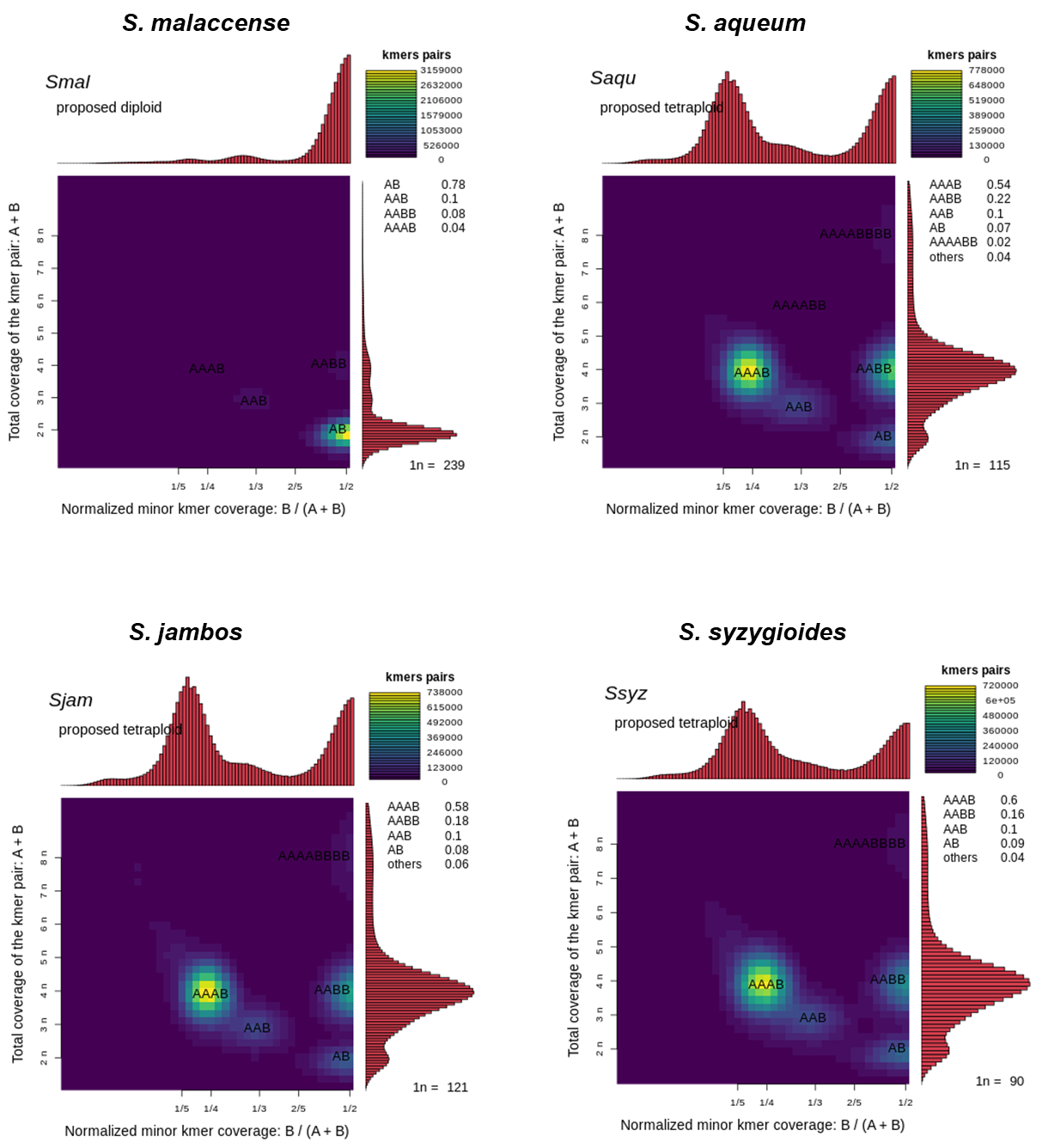


**Supplemental Figure 2.** Smudgeplot results for the *S. malaccense*, *S. aqueum*, *S. jambos*, and *S. syzygioides* genomes.

**
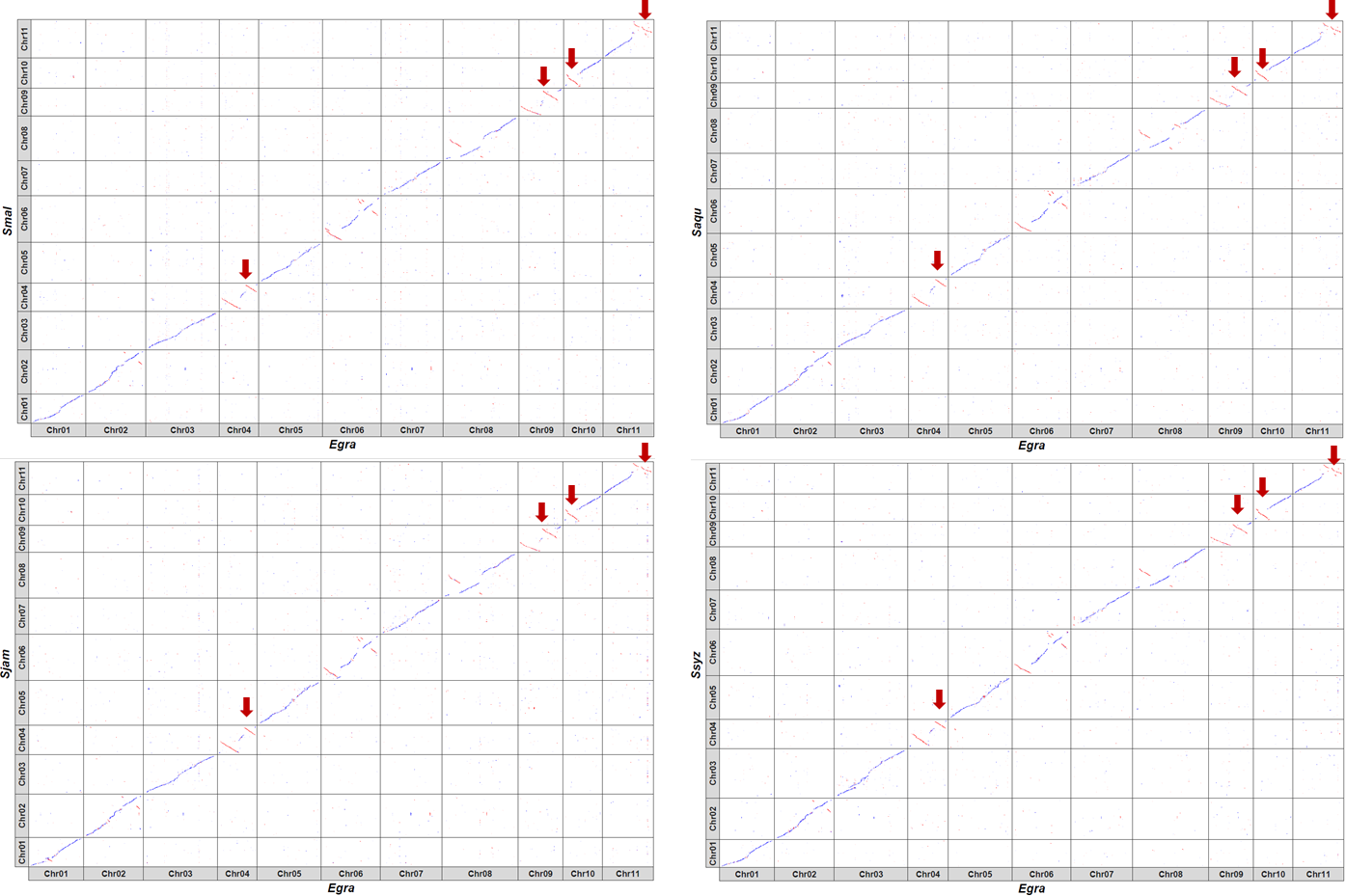
**

**Supplemental Figure 3.** DNA alignment of the 11 chromosomes of *S. malaccense* (Smal)*, S. aqueum* (Saqu)*, S. jambos* (Sjam), and *S. syzygioides* (Ssyz) versus *E. grandis* (Egra) chromosomes. Aligned DNA sequences and intrachromosomal rearrangements are shown in blue and red, respectively. Red arrows indicate regions where similar large inversions were detected between *S. aromaticum* and *E. grandis* and *E. grandis* and *Corymbia citriodora*.

**
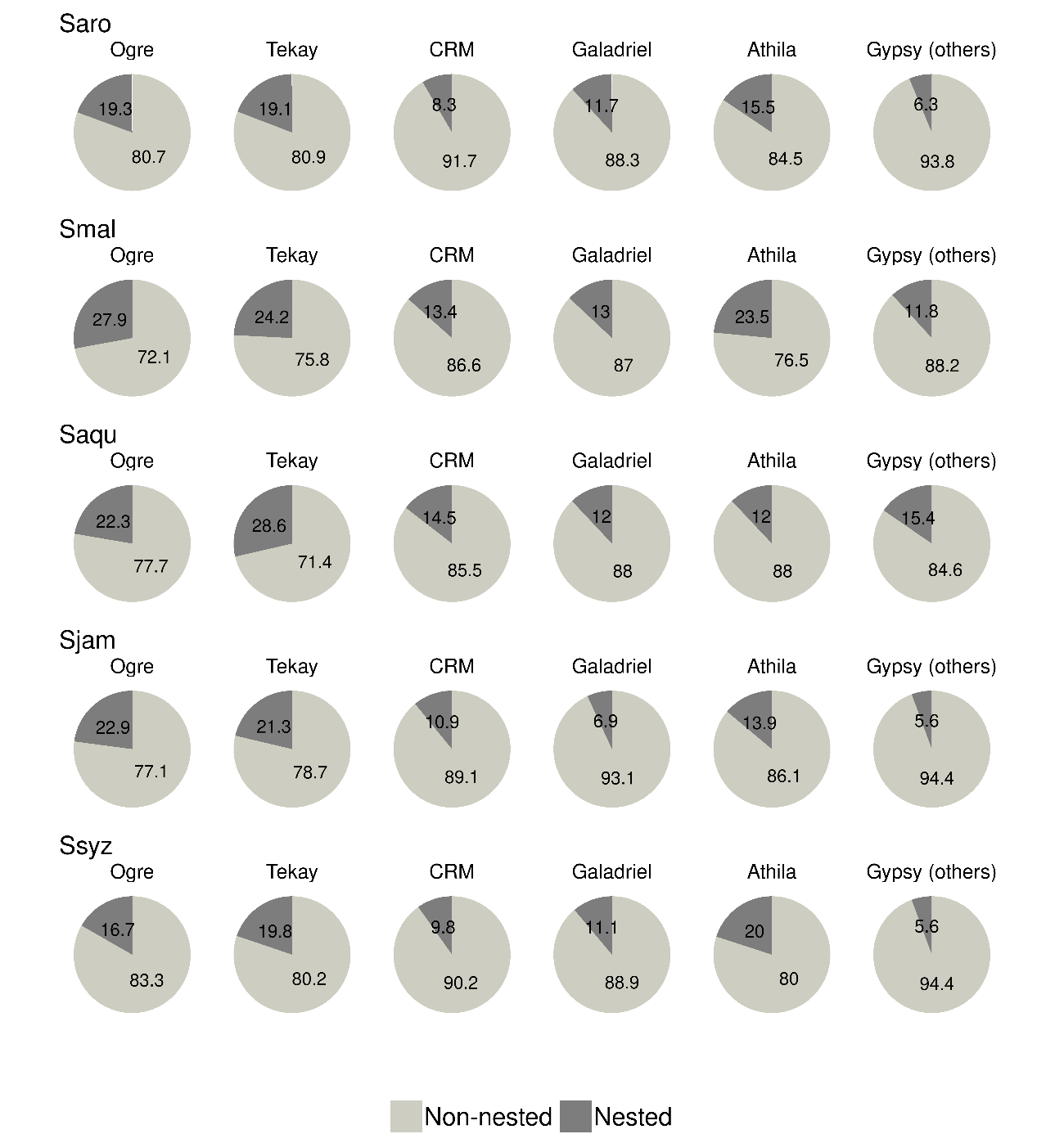
**

**Supplemental Figure 4.** The proportion of non-nested and nested elements in the Gypsy lineages of *S. aromaticum* (Saro), *S. malaccense* (Smal), *S. aqueum* (Saqu), *S. jambos* (Sjam), and *S. syzygioides* (Ssyz). The Gypsy (others) group comprises the lineages non-chromo-outgroup, Reina, Retand, tatIII, and elements Gypsy for which no lineages were assigned to.

**
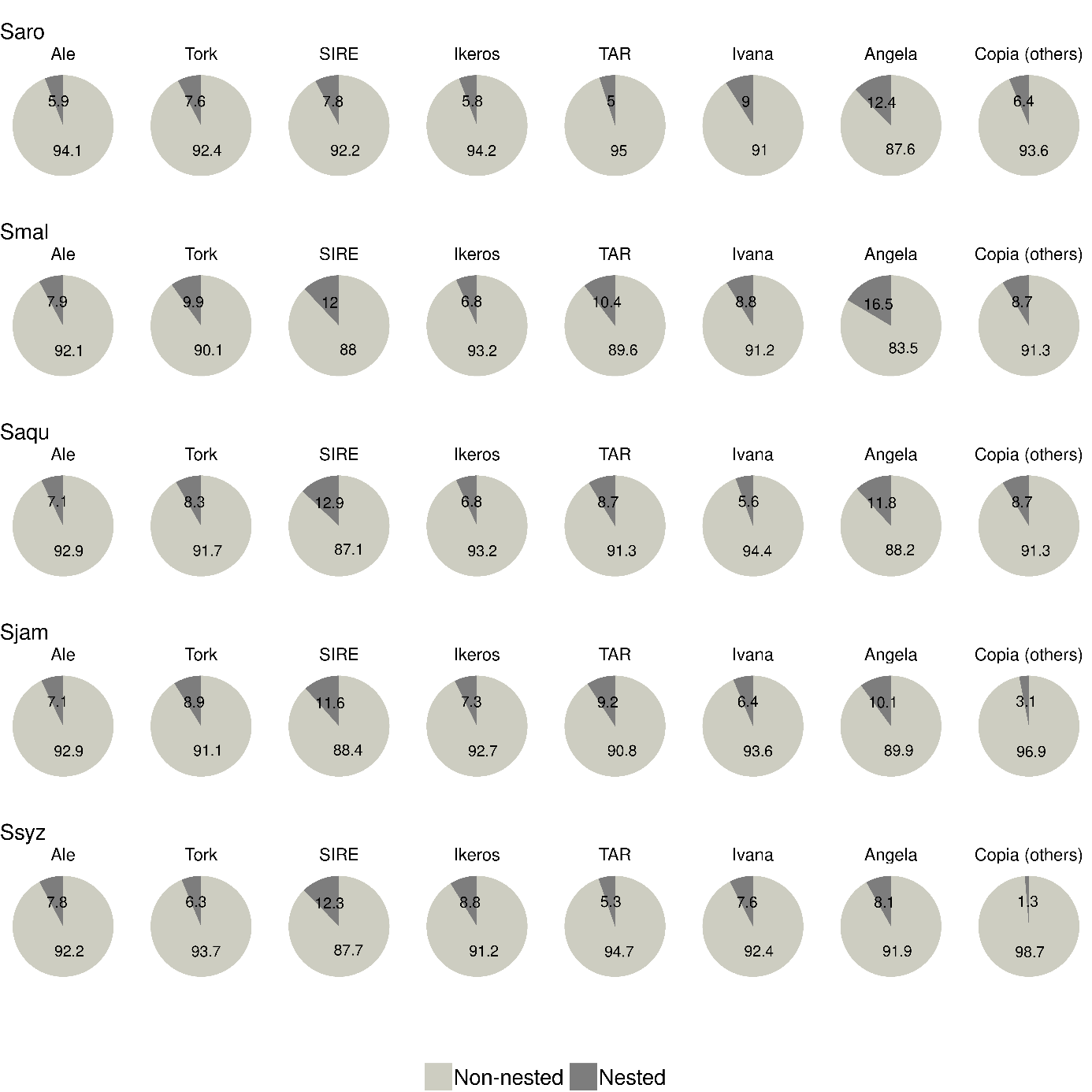
**

**Supplemental Figure 5.** The proportion of non-nested and nested elements in the Copia lineages of *S. aromaticum* (Saro), *S. malaccense* (Smal), *S. aqueum* (Saqu), *S. jambos* (Sjam), and *S. syzygioides.* Copia (others) group comprises the lineages Alesia, Bianca, Gymco-I, Gymco-IV, Gymco-II, Osser.

**Supplemental Table 1.** Illumina sequencing summary. Number of Illumina short PE reads used as input for the genome profiling (using GenomeScope 2.0 and Smudgeplot), the ONT reads error correction, and scaffolding steps.

|  | Species name | Number of reads | Total bases (bp) |
| --- | --- | --- | --- |
| Genome profiling and ONT^1^ reads error correction |  |  |  |
|  | *S. malaccense* | 1,820,074,174 | 268,411,454,544 |
|  | *S. aqueum* | 1,596,353,896 | 235,653,512,752 |
|  | *S. jambos* | 1,787,425,820 | 264,313,064,388 |
|  | *S. syzygioides* | 1,252,027,700 | 184,705,961,534 |
| Scaffolding (Hi-C reads) |  |  |  |
|  | *S. malaccense* | 1,091,335,212 | 162,074,310,050 |
|  | *S. aqueum* | 975,059,968 | 138,895,360,360 |
|  | *S. jambos* | 1,197,724,004 | 178,804,480,944 |
|  | *S. syzygioides* | 1,373,567,222 | 204,377,985,402 |

^1^ ONT = Oxford Nanopore Technologies

**Supplemental Table 2.** ONT sequencing summary. ONT raw reads were first cleaned to discard reads shorter than 5,000 bp or with quality scores lower than 9.

|  | Species name | Number of reads | Total bases (bp) |
| --- | --- | --- | --- |
| ONT cleaned reads | | | |
|  | *S. malaccense* | 8,252,824 | 202,704,475,235 |
|  | *S. aqueum* | 9,493,220 | 224,762,123,191 |
|  | *S. jambos* | 9,648,395 | 219,016,140,340 |
|  | *S. syzygioides* | 6,944,019 | 194,208,708,693 |

**Supplemental Table 3.** Genome assemblies’ quality assessment using BUSCO version 5.4.4 - dataset: eudicots_odb10 (n = 2326) in mode genome, transcriptome, and Protein. Complete BUSCOs (C), Complete and single copy BUSCOs (S), Complete and duplicated BUSCOs (D), Fragmented BUSCOs (F), Missing BUSCOs (M).

|  | % C | % S | % D | % F | % M |
| --- | --- | --- | --- | --- | --- |
| BUSCO genome  (Contigs before haplotig removal) |  |  |  |  |  |
| *S. malaccense* | 98.7 | 5.1 | 93.6 | 0.5 | 0.8 |
| *S. aqueum* | 98.7 | 4.3 | 94.4 | 0.6 | 0.7 |
| *S. jambos* | 98.7 | 3.4 | 95.3 | 0.6 | 0.7 |
| *S. syzygioides* | 98.9 | 1.8 | 97.1 | 0.5 | 0.6 |
|  |  |  |  |  |  |
| BUSCO genome  (Contigs after haplotig removal) |  |  |  |  |  |
| *S. malaccense* | 98.1 | 94.8 | 3.3 | 0.7 | 1.2 |
| *S. aqueum* | 98 | 93.2 | 4.8 | 0.7 | 1.3 |
| *S. jambos* | 97.9 | 93.9 | 4 | 0.7 | 1.4 |
| *S. syzygioides* | 98 | 91.9 | 6.1 | 0.7 | 1.3 |
| BUSCO genome  (Final assembly) |  |  |  |  |  |
| *S. aromaticum* | 98.1 | 95.9 | 2.2 | 0.9 | 1 |
| *S. malaccense* | 98.1 | 94.8 | 3.3 | 0.6 | 1.3 |
| *S. aqueum* | 98 | 93.5 | 4.5 | 0.7 | 1.3 |
| *S. jambos* | 98 | 94.4 | 3.6 | 0.7 | 1.3 |
| *S. syzygioides* | 98.2 | 92.7 | 5.5 | 0.6 | 1.2 |
|  |  |  |  |  |  |
| BUSCO transcriptome |  |  |  |  |  |
| *S. aromaticum* | 95 | 47.9 | 47.1 | 1 | 4 |
| *S. malaccense* | 93.5 | 55.8 | 37.7 | 1.9 | 4.6 |
| *S. aqueum* | 91.9 | 52.8 | 39.1 | 2.8 | 5.3 |
| *S. jambos* | 92.6 | 55.5 | 37.1 | 2.3 | 5.1 |
| *S. syzygioides* | 92.7 | 52.6 | 40.1 | 2.8 | 4.5 |
|  |  |  |  |  |  |
| BUSCO protein |  |  |  |  |  |
| *S. aromaticum* | 93.7 | 59.9 | 33.8 | 1.5 | 4.8 |
| *S. malaccense* | 90.9 | 63.6 | 27.3 | 2.6 | 6.5 |
| *S. aqueum* | 89.3 | 61.8 | 27.5 | 3.4 | 7.3 |
| *S. jambos* | 90.1 | 63.1 | 27 | 3.3 | 6.6 |
| *S. syzygioides* | 89.8 | 60.5 | 29.3 | 3.5 | 6.7 |

**Supplemental Table 4**. Chromosome length (bp) of *S. aromaticum*, *S. malaccense*, *S. aqueum*, *S. jambos*, *S. syzygioides*, and *S. grande*.

| Chromosome | *S. aromaticum* | | *S. malaccense* | | *S. aqueum* | | *S. jambos* | | *S. syzygioides* | | *S. grande* |
| --- | --- | --- | --- | --- | --- | --- | --- | --- | --- | --- | --- |
| Chr01 | 28,385,693 | 3,119,1675 | | 28,960,203 | | 30,275,079 | | 29,286,511 | | 30,130,455 | |
| Chr02 | 36,857,792 | 46,997,391 | | 43,113,907 | | 44,223,308 | | 43,522,896 | | 41,423,945 | |
| Chr03 | 35,418,074 | 40,588,123 | | 38,810,027 | | 40,716,907 | | 52,151,277 | | 39,560,356 | |
| Chr04 | 27,127,432 | 30,283,374 | | 30,431,105 | | 29,830,967 | | 30,781,539 | | 27,788,763 | |
| Chr05 | 38,056,112 | 43,125,459 | | 41,576,880 | | 45,836,902 | | 46,132,905 | | 41,797,999 | |
| Chr06 | 42,993,409 | 49,125,077 | | 42,880,593 | | 47,627,361 | | 48,951,463 | | 43,109,847 | |
| Chr07 | 33,360,290 | 37,421,253 | | 33,918,699 | | 37,171,488 | | 40,604,280 | | 35,655,372 | |
| Chr08 | 43,763,418 | 47,424,843 | | 43,361,234 | | 47,089,386 | | 45,885,160 | | 43,366,331 | |
| Chr09 | 23,380,519 | 29,684,569 | | 24,382,885 | | 27,657,921 | | 26,538,911 | | 23,649,235 | |
| Chr10 | 26,565,453 | 32,139,982 | | 26,467,591 | | 31,499,097 | | 28,595,216 | | 28,611,675 | |
| Chr11 | 31,862,996 | 41,026,473 | | 32,633,549 | | 33,694,566 | | 32,377,069 | | 32,526,569 | |
| Total | 367,771,188 | 429,008,219 | | 386,536,673 | | 415,622,982 | | 424,827,227 | | 387,620,547 | |

**Supplemental Table 5.** List of genes encoding for putative eugenol synthase (EGS) and their position on the chromosome (Chr) of *S. malaccense*, *S. aqueum*, *S. jambos*, and *S. syzygioides*.

| Species | Gene | Protein | Annotation | Chr | Start (bp) | End (bp) |
| --- | --- | --- | --- | --- | --- | --- |
| *S. malaccense* | Smal007380 | EGS1 | eugenol synthase 1-like | Chr10 | 21,561,954 | 21,566,251 |
| *S. aqueum* | Saqu028928 | EGS1 | eugenol synthase 1-like | Chr10 | 17,568,090 | 17,569,992 |
| *S. aqueum* | Saqu028929 | EGS2 | eugenol synthase 1-like | Chr10 | 17,600,976 | 17,604,578 |
| *S. jambos* | Sjam030623 | EGS1 | eugenol synthase 1-like | Chr10 | 22,078,314 | 22,082,922 |
| *S. jambos* | Sjam030624 | EGS2 | eugenol synthase 1-like | Chr10 | 22,085,199 | 22,088,927 |
| *S. jambos* | Sjam030625 | EGS3 | eugenol synthase 1-like | Chr10 | 22,090,133 | 22,092,406 |
| *S. syzygioides* | Ssyz031161 | EGS1 | eugenol synthase 1-like | Chr10 | 19,161,196 | 19,165,512 |
| *S. syzygioides* | Ssyz031162 | EGS2 | eugenol synthase 1-like | Chr10 | 19,167,723 | 19,170,887 |
| *S. syzygioides* | Ssyz002596 | EGS3 | eugenol synthase 1-like | Chr11 | 30,519,613 | 30,522,208 |

**Supplemental Table 6**. Summary of repeats elements identified in the genome assembly of *S. malaccense*.

| Class | Superfamily | Lineage | Count | Length (bp) | Percentage of assembly length |
| --- | --- | --- | --- | --- | --- |
| Retrotransposons |  |  | 8515 | 96,086,564 | 22.35% |
|  | LTR^1^ Copia |  | 3435 | 31,769,467 | 7.39% |
|  |  | Ale | 835 | 7,018,745 | 1.63% |
|  |  | Alesia | 39 | 373,075 | 0.09% |
|  |  | Angela | 139 | 1,273,090 | 0.30% |
|  |  | Bianca | 34 | 264,888 | 0.06% |
|  |  | Gymco-II | 1 | 10,679 | 0.00% |
|  |  | Ikeros | 529 | 4,877,541 | 1.13% |
|  |  | Ivana | 329 | 2,369,071 | 0.55% |
|  |  | SIRE | 523 | 5,747,540 | 1.34% |
|  |  | TAR | 280 | 2,466,390 | 0.57% |
|  |  | Tork | 708 | 7,002,611 | 1.63% |
|  |  | Unknown | 18 | 365,837 | 0.09% |
|  | LTR Gypsy |  | 4995 | 62,668,430 | 14.58% |
|  |  | Athila | 98 | 1,025,135 | 0.24% |
|  |  | CRM | 619 | 4,537,097 | 1.06% |
|  |  | Galadriel | 46 | 459,408 | 0.11% |
|  | non-chromo-outgroup | | 1 | 6945 | 0.002% |
|  |  | Ogre | 2382 | 37,168,658 | 8.65% |
|  |  | Reina | 8 | 58,597 | 0.01% |
|  |  | Tekay | 1833 | 19,326,726 | 4.50% |
|  |  | Unknown | 8 | 85,864 | 0.02% |
|  | LTR Unknown |  | 85 | 1,648,667 | 0.38% |
| DNA transposons | MITE^2^ |  | 5710 | 594,020 | 0.14% |
|  | Helitron |  | 1384 | 39,200 | 0.01% |
| Tandem repeats |  |  | 164,955 | 4,058,239 | 0.94% |
| Repeats elements (unclassified) |  |  | 149,758 | 84,138,834 | 19.57% |
| Total |  |  | 3303,22 | 184,916,857 | 43.02% |

^1^ LTR = Long Terminal Repeat

^2^ MITE = Miniature Inverted-repeat Transposable Elements

**Supplemental Table 7**. Summary of repeats elements identified in the genome assembly of *S. aqueum*

| Class | Superfamily | Lineage | Count | Length (bp) | Percentage of assembly length |
| --- | --- | --- | --- | --- | --- |
| Retrotransposons |  |  | 6541 | 74,914,968 | 19.12% |
|  | LTR^1^ Copia |  | 2743 | 25,148,956 | 6.42% |
|  |  | Ale | 839 | 7,286,400 | 1.86% |
|  |  | Alesia | 38 | 264,985 | 0.07% |
|  |  | Angela | 97 | 850,005 | 0.22% |
|  |  | Bianca | 54 | 400,622 | 0.10% |
|  |  | Gymco-I | 1 | 9997 | 0.00% |
|  |  | Ikeros | 374 | 3,550,808 | 0.91% |
|  |  | Ivana | 290 | 1,997,761 | 0.51% |
|  |  | SIRE | 305 | 3,478,327 | 0.89% |
|  |  | TAR | 272 | 2,452,073 | 0.63% |
|  |  | Tork | 460 | 4,666,215 | 1.19% |
|  |  | Unknown | 13 | 191,763 | 0.05% |
|  | LTR Gypsy |  | 3710 | 48,141,612 | 12.28% |
|  |  | Athila | 52 | 577,550 | 0.15% |
|  |  | CRM | 350 | 2,961,734 | 0.76% |
|  |  | Galadriel | 25 | 305,966 | 0.08% |
|  | non-chromo-outgroup | | 1 | 8977 | 0.002% |
|  |  | Ogre | 1829 | 27,773,931 | 7.09% |
|  |  | Reina | 10 | 81,745 | 0.02% |
|  |  | Tekay | 1441 | 16,386,086 | 4.18% |
|  |  | Unknown | 2 | 45,623 | 0.01% |
|  | LTR Unknown |  | 88 | 1,624,400 | 0.41% |
| DNA transposons | MITE^2^ |  | 4,034 | 580,151 | 0.15% |
|  | Helitron |  | 1198 | 33,858 | 0.01% |
| Tandem repeats |  |  | 147,952 | 3,621,605 | 0.92% |
| Repeats elements (unclassified) |  |  | 139,832 | 82,869,853 | 21.15% |
| Total |  |  | 299,557 | 162,020,435 | 41.34% |

^1^ LTR = Long Terminal Repeat

^2^ MITE = Miniature Inverted-repeat Transposable Elements

**Supplemental Table 8.** Summary of repeats elements identified in the genome assembly of *S. jambos.*

| Class | Superfamily | Lineage | Count | Length (bp) | Percentage of assembly length |
| --- | --- | --- | --- | --- | --- |
| Retrotransposons |  |  | 7393 | 77,407,268 | 18.16% |
|  | LTR^1^ Copia |  | 3402 | 30,040,740 | 7.05% |
|  |  | Ale | 876 | 7,176,078 | 1.68% |
|  |  | Alesia | 42 | 378,260 | 0.09% |
|  |  | Angela | 82 | 822,474 | 0.19% |
|  |  | Bianca | 45 | 330,379 | 0.08% |
|  |  | Ikeros | 607 | 5,527,257 | 1.30% |
|  |  | Ivana | 273 | 1,910,486 | 0.45% |
|  |  | SIRE | 468 | 5,013,322 | 1.18% |
|  |  | TAR | 336 | 2,815,655 | 0.66% |
|  |  | Tork | 661 | 5,871,859 | 1.38% |
|  |  | Unknown | 12 | 194,970 | 0.05% |
|  | LTR Gypsy |  | 3858 | 45,090,450 | 10.58% |
|  |  | Athila | 73 | 803,315 | 0.19% |
|  |  | CRM | 544 | 3,657,979 | 0.86% |
|  |  | Galadriel | 209 | 1,213,756 | 0.28% |
|  | non-chromo-outgroup | | 4 | 72,530 | 0.02% |
|  |  | Ogre | 1258 | 20,157,864 | 4.73% |
|  |  | Reina | 9 | 55,716 | 0.01% |
|  |  | Retand | 1 | 10,992 | 0.00% |
|  |  | TatIII | 1 | 3450 | 0.00% |
|  |  | Tekay | 1754 | 19,067,733 | 4.47% |
|  |  | Unknown | 5 | 47,115 | 0.01% |
|  | LTR Unknown |  | 133 | 2,276,078 | 0.53% |
| DNA transposons | MITE^2^ |  | 10,323 | 1,131,284 | 0.27% |
|  | Helitron |  | 1652 | 46,555 | 0.01% |
| Tandem repeats |  |  | 165,419 | 4,055,420 | 0.95% |
| Repeats elements (unclassified) |  |  | 160,407 | 97,923,066 | 22.98% |
| Total |  |  | 345,194 | 180,563,593 | 42.37% |

^1^ LTR = Long Terminal Repeat

^2^ MITE = Miniature Inverted-repeat Transposable Elements

**Supplemental Table 9**. Summary of repeats elements identified in the genome assembly of *S. syzygioides.*

| Class | Superfamily | Lineage | Count | Length (bp) | Percentage of assembly length |
| --- | --- | --- | --- | --- | --- |
| Retrotransposons |  |  | 7144 | 73,171,928 | 16.97% |
|  | LTR^1^ Copia |  | 3348 | 30,532,813 | 7.08% |
|  |  | Ale | 885 | 7,387,426 | 1.71% |
|  |  | Alesia | 31 | 229,884 | 0.05% |
|  |  | Angela | 88 | 788,496 | 0.18% |
|  |  | Bianca | 29 | 267,998 | 0.06% |
|  |  | Ikeros | 520 | 5,028,520 | 1.17% |
|  |  | Ivana | 267 | 1,975,430 | 0.46% |
|  |  | Osser | 1 | 11,413 | 0.00% |
|  |  | SIRE | 429 | 4,953,376 | 1.15% |
|  |  | TAR | 307 | 2,767,941 | 0.64% |
|  |  | Tork | 774 | 6,746,234 | 1.56% |
|  |  | Unknown | 17 | 376,095 | 0.09% |
|  | LTR Gypsy |  | 3659 | 40,369,474 | 9.36% |
|  |  | Athila | 52 | 535,287 | 0.12% |
|  |  | CRM | 497 | 3,683,608 | 0.85% |
|  |  | Galadriel | 117 | 693,929 | 0.16% |
|  | non-chromo-outgroup | | 3 | 23,449 | 0.01% |
|  |  | Ogre | 844 | 13,492,111 | 3.13% |
|  |  | Reina | 13 | 93,044 | 0.02% |
|  |  | Tekay | 2128 | 21,795,136 | 5.06% |
|  |  | Unknown | 5 | 52,910 | 0.01% |
|  | LTR Unknown |  | 137 | 2,269,641 | 0.53% |
| DNA transposons | MITE^2^ |  | 6525 | 667,396 | 0.15% |
|  | Helitron |  | 1579 | 44,821 | 0.01% |
| Tandem repeats |  |  | 157,919 | 3,834,074 | 0.89% |
| Repeats elements (unclassified) |  |  | 177,217 | 106,284,882 | 24.66% |
| Total |  |  | 350,384 | 184,003,101 | 42.68% |

^1^ LTR = Long Terminal Repeat

^2^ MITE = Miniature Inverted-repeat Transposable Elements
